# Case of Successful Removal of Foreign Body in the Throat by Tracheotomy

**Published:** 1863-09

**Authors:** William M. McDowell

**Affiliations:** Canton, Fulton Co., Ill.


Case of Successful Removal of Foreign Body in the Throat by Tracheotomy.—*By William M. McDowell, M. D., Canton, Fulton Co., Ill.*—Sarah Hanks, six years old next August, and daughter of Mrs. Ladicia Hanks, a widow, of Canton, Illinois, while engaged in recreation at noon with her school-playmates on Wednesday, the 18th of March, 1863, was in the act of laughing heartily, with her head inclined backwards, with a large grain of corn in her mouth; during inspiration the corn passed through the glottis into the trachea, giving rise to the usual alarming symptoms. She immediately fell down, with distressing indications of suffocation, frightening her associates, and inducing them to think she was dying. She was soon conveyed home, and her mother sent for her physician—a Hahnemannian. The means he employed were confined to the administration of sugar-pellicles, hopeful, we suppose, that the grain might be ejected in coughing, and his fanciful agents have the credit of causing its expulsion. The mother becoming apprehensive for the safety of her child from these frequent paroxysms of coughing, called in another medical adviser, who confided in the administration of errhines and emetics. Being ignorant of the necessary procedure herself, and influenced by the pernicious counsels of others, she permitted the little sufferer to remain in this distressing condition until the 31st of March—the fourteenth day of her troublesome affliction—when I was consulted in regard to her condition, and recommended extraction, as the only probable salvation, which, from the ravages which had been made, was apparently a forlorn hope. The mother informed me she was frequently advised not to permit the operation, as it was extremely hazardous, but, as the child was failing so rapidly, she was willing it should be resorted to, and gave a deplorable history of her condition from the time the corn entered the trachea—That her voice soon failed to a hoarse, low, wheezing whisper; she could not recline in bed, but had to be held up in a sitting posture; was thirsty, and drank frequently, very little at a time, and she was confident did not take more nourishment than two table-spoonsfull of rice, and did not sleep at all during this time; was so irritable when spoken to as to strike at the person addressing her. Paroxysms of coughing and dyspnœa were frequently renewed, and continued for several hours at a time. The child, on the 31st, was extremely emaciated and prostrated; pulse feeble and accelerated; eyes dull and languid; face of a pale, dusky appearance. Drs. Ingersoll, Martin, Fleming and Searles were invited in, and one of the number decided against the operation, as in his opinion there was no prospect of saving the child. The remaining four of us decided that the prostration and symptoms of marasmus left scarcely a hope, and, as tracheotomy afforded the only chance, the child should have this, dim as it appeared. Being very irritable and excitable, she was put under the influence of chloroform, and an incision was made just below the cricoid cartilage in a perpendicular direction, approximating the top of the sternum, dividing the superficial and deep fasciæ and cellular structure at the junction of the sterno-hyoid muscles; then, with the point and handle of the knife their connections were separated, and the trachea laid bare. With a small scalpel two rings were opened. On this there followed a discharge of muco-purulent matter tinged with blood. We waited for half an hour, expecting every moment the corn to be ejected, when a director was introduced, and the back of the scalpel glided along the groove, and another ring was severed. The director was moved over the sides and superior portion of the windpipe, and withdrawn with the handle depressed upon the sternum, pressing the instrument moderately in contact with the anterior portion as it was extracted, which brought with it a considerable portion of adventitious membrane and the grain of corn. Laryngo-trachitis had evidently been created by this foreign body, resulting in a pseudo-membranous formation, and the corn may have been resting on it or entangled in it.

But little hemorrhage was encountered, and as soon as it entirely ceased the aperture was closed with strips of adhesive plaster. The aperture is now closed and healed. The result of this operation was unexpectedly satisfactory and successful. Repose and a vigorous appetite rapidly developed the child's physical condition, and those who had seen her enfeebled and emaciated, in a few days after her relief did not know her, recovery being so speedy. The child is completely restored to health. In connection with this case we may contemplate two important achievements worthy of favorable consideration.

If we should be called to a similar case, when from circumstances over which we have had no control, valuable time has been lost, and the case is apparently beyond the prospect of hope, we should have no hesitation in opening the trachea. The case just narrated affords additional corroborative testimony demonstrating the advantage of tracheotomy for the removal of preternatural membranous formation in laryngo-trachitis.—*American Medical Times*, Aug. 12, 1863.

